# A Plea for Raising War Office Payments with a Flat Rate

**Published:** 1917-08-18

**Authors:** 


### WARRIOR PATIENTS IN THE VOLUNTARY HOSPITALS.

### A Plea for Raising War Office Payments with a Flat Rate.

In the issue of The Hospital of August 4, attention was called in a review of the statistics recently published by the Red Cross Society to the immensity of the work performed in 982 V.A.D. hospitals. It was shown that the cost of maintenance was approximately £2,000,000. An examination of the figures set out with admirable clearness in the report shows that the average cost of maintenance per patient per day in the combined hospitals was over 3s. 6d., but it is pointed out that not in every case have rents and rates been brought into account, that the figures take no account of the voluntary services rendered in many hospitals by the *personnel*, and that in certain instances the salaries of nurses supplied by Headquarters or by local branches of the B.R.C. have not in every case been included. The figures to this extent do not afford a perfect comparison with the cost of maintenance in the larger institutions, but they do throw a good deal of illumination on the cost of hospital administration generally.

The Hospital has on several occasions adduced that the War Office made a good bargain when at the beginning of the war they entered into negotiations and agreement with the voluntary hospitals to receive military patients. The maximum rate of remuneration was 4s. per day. Owing to the greatly enhanced price of all purchasable commodities, and especially food, dressings, appliances, fuel, bedding, and linen (not to mention the increased salaries of resident nurses and attendants), a general feeling exists that the War Office should increase the maximum grant. A great difference exists in the cost of treatment of patients received by direct convoys, for which active medical and surgical treatment and costly dressings are called for, and in the treatment of convalescent cases, for which the V.A.D. hospitals provide so efficiently.

The fact is, that the rates received by the voluntary hospitals bear little relation to the actual cost of treatment. Some idea of this actual cost may be gathered from a reference to page 136 of the B.R.C. Report, which gives the figures in the only two voluntary hospitals from which returns are published—namely, the Liverpool Royal Infirmary and the Liverpool Royal Southern Hospital, where the cost per day is shown to be 4s. 9d. and 4s. 8d. respectively. The voluntary institutions have always been anxious to render every possible assistance to the country, and their co-operation in the supply of doctors and nurses for war service has often been eulogised. For these reasons it would be a graceful act on the part of the War Office spontaneously to increase the remuneration to an average rate of 4s. 6d., or, better still, 5s. per diem. Justice calls further for the same concessions to be made that are made to the V.A.D. hospitals, by the grant of a flat rate for unoccupied beds. Such beds are, of course, a constant charge upon institutions for wear and tear, washing and upkeep. The voluntary hospitals have continuously provided the War Office with the fullest aid in accommodation and *personnel*. The consideration of these points by the War Office is admittedly justly due. Increased grants are justified by the circumstances, and they may spontaneously and properly be offered without direct application by the hospitals. The prompt way in which the accounts of the institution are passed and payment made by the various Commands is generally appreciated.

